# Forewing structure of the solitary bee *Osmia bicornis* developing on heavy metal pollution gradient

**DOI:** 10.1007/s10646-017-1831-2

**Published:** 2017-07-08

**Authors:** Hajnalka Szentgyörgyi, Dawid Moroń, Anna Nawrocka, Adam Tofilski, Michał Woyciechowski

**Affiliations:** 1 Department of Pomology and Apiculture, University of Agriculture in Kraków, Al. 29. Listopada 54, Kraków, 31-425 Poland; 2 Institute of Systematics and Evolution of Animals, Polish Academy of Sciences, Sławkowska 17, Kraków, 31-016 Poland; 3 Institute of Environmental Sciences, Jagiellonian University, Gronostajowa 7, Kraków, 30-387 Poland

**Keywords:** *Osmia bicornis*, Pollution, Heavy metals, Wing asymmetry

## Abstract

Wild bees in natural conditions can develop under various environmental stressors. Heavy metal pollution of the environment is one of the most widely studied stressors in insects, yet its effect is poorly described in bees. We have measured how pollution of the environment along a zinc, cadmium and lead contamination gradient in Poland affects bee development, using red mason bees (*Osmia bicornis*) as a model and their forewing asymmetry measures to assess possible developmental instabilities. We have also described wing asymmetry measures in the red mason bee—an important managed pollinator species—for the first time. The development of bee larvae in a contaminated environment did not affect forewing asymmetry measures, but it did lead to a negative correlation of wing size with contamination in females. Bees also showed a clear change in their asymmetry measures between various seasons, suggesting other, unknown environmental factors affecting wing asymmetry more than pollution. Sexes were found to have different forewing shape and size, larger females having larger forewings than the smaller males. The direction of size asymmetry was in favour of the left side in both sexes and also shape differences between the left and right wings showed similar tendencies in males and females. The levels of forewing shape and size asymmetry were smaller in females, making them the more symmetrical sex.

## Introduction

Bilateral organisms are not perfectly symmetrical. Deviations from perfect symmetry can appear either in a regular or irregular fashion. Regularly appearing, left-right asymmetry in favour of one side (a paired organ or body part being regularly larger, longer, wider etc. on a certain side) is called directional asymmetry (van Valen 1962) and is usually characteristic of a species or even one sex in a given species. A typical example of directional asymmetry is right-handedness in humans. Although part of the population is left-handed, significantly a larger proportion of humans are right-handed. Besides directional asymmetry, randomly appearing and normally distributed (appearing on both sides) small deviations from perfect symmetry, called fluctuating asymmetry (FA), can also be observed, and is suggested that these arise due to developmental instability and random environmental effects on the developing organism (Mather 1953; van Valen 1962; Palmer and Strobeck 1992; Palmer 1994). It is often assumed that more pronounced developmental instability is causing greater degrees of asymmetry in the organism. However, this correlation was not confirmed in many species and traits, therefore asymmetry cannot be treated as a direct measure of developmental instability (Palmer and Strobeck 2003). Nonetheless, asymmetry is often used in describing the effects of various stressors like changes of temperature (Jones et al. 2005), nutritional conditions during development (Grønkjær and Sand 2003) or toxicity (Graham et al. 1993). Geometric morphometric analysis of the wings in flying insects is gaining more and more attention as a method of identifying species (Lyra et al. 2010; Francoy et al. 2012), subspecies (Tofilski 2008), populations (Lima et al. 2014) or even genetic lineages (Francoy et al. 2011) with promising results. Furthermore, analysis of wing asymmetry is also being tested as a possible tool to assess the level of developmental instability caused by various stressors like inbreeding (Brückner 1976), hybridization (Smith et al. 1997) or starvation (Szentgyörgyi et al. 2016) with varying results.

Pollution of the environment with substances of anthropogenic origin like pesticides or heavy metals pose a clear threat to species developing in polluted areas. Pesticide exposure besides clear acute toxicity for the target species (pests) have also measurable sublethal effects often for other beneficial species like e.g. bees. Developmental malformations, weight reduction suppression of gland development are among the documented sublethal effects of pesticide exposure of bee larvae to pesticides (Desneux et al. 2007) but also changes in fluctuating asymmetry of body parts was reported by Ondo Zue Abaga and his colleagues (2011). Besides pesticides, heavy metals are also widely studied environmental stressors affecting the development and functioning of the organism. Some are essential to the biochemical and physiological functioning of the organism (e.g. zinc, iron, copper), but become toxic when given in excess. Others, so called xenobiotics, are toxic in all amounts (e.g. lead, mercury or plutonium) beyond their natural background level (Newman and Clements 2008). Both xenobiotics and essential trace metals (when given in excess) can weaken an organism by changing the conformation or causing the denaturation of enzymes (Newman and Clements 2008). In ants heavy metal contamination causes, for example, a generally weaker immune response (Sorvari et al. 2007), suggesting that pathogens and parasites can more easily enter and induce heavy infections in individuals (Galloway and Depledge 2001). Heavy metals also affect the developing organism. Lead poisoning in humans is well described and known to affect pregnancy outcomes and causes foetal growth retardation (Bellinger 2005), while a foetus with mercury poisoning has severe disabilities (EPA 2014). In invertebrates heavy metal contamination was shown to alter early embryonic development (Gopalakrishnan et al. 2008) or even be lethal for the embryo (Calabrese and Nelson 1974). The impact of heavy metal pollution on wild bees was only described in a few contamination gradients (Poland and England: Moroń et al. 2012, 2014; Poland and Russia: Szentgyörgyi et al. 2011), although it is well studied in other groups of invertebrates and the general effects at the ecosystem level are well documented (for a review see Tyler et al. 1989). Heavy metals emitted to the environment are efficient at settling and accumulating in soil and litter due to their affinity for clay particles and organic substances (Walker et al. 2012), therefore soil living organisms or those that are coming into regular contact with soil and litter (like ground-nesting or soil using bee species) can suffer from airborne and soil accumulated contamination.

Solitary bees, thanks to their role in crop pollination and the possibility of managing them successfully (Bosch and Kemp 2002; Krunić and Stanisavljević 2006), are often used in ecological studies describing the effects of environmental stressors. The red mason bee (*Osmia bicornis* Panzer), a widespread European species, is particularly widely studied concerning its general biology, nesting and development (Raw 1972; Radmacher and Strohm 2010; Seidelmann et al. 2009; Szentgyörgyi and Woyciechowski 2013; Wasielewski et al. 2011; Kierat et al. 2017a). Red mason bees, although considered rather as a species that nests above ground, can come into direct contact with both airborne contamination during foraging and pollution accumulated in the soil due to using soil for building the walls separating their cells containing offspring (Bosch et al. 1993). Heavy metal pollution during development was already shown to negatively affect the survival and body mass at emergence of red mason bee offspring (Moroń et al. 2014).

Here we have analyzed red mason bees developing along a heavy metal pollution gradient in Poland contaminated mainly with zinc, cadmium, and lead to verify if an increased concentration of heavy metals in the environment can cause greater asymmetry of their forewing venation. For the first time, we have also described the pattern of directional asymmetry of forewings in this species.

## Materials and methods

### Field sites and pollution measurements

The study was carried out in the vicinity of the zinc smelter operating near Olkusz (50°16′38″N, 19°28′17″E) in Lesser Poland Voivodship in Poland since 1967. The smelter mainly emitted zinc (Zn), lead (Pb) and cadmium (Cd) to the environment (Stone et al. 2002). In the first year of the study, five sites were selected (OM2, 3, 4, 6, 7) based on the concentration of metals measured in the topsoil by Stefanowicz et al. (2008), while in the following two years, two more were added and altogether seven sites (OM1, 2, 3, 4, 5, 6, 7) were selected along the pollution gradient. OM1 was the most and OM7 the least polluted site. Sites were more than 1 km apart and had similar, poor, sandy soils, a size of approx. 20 ha and a landscape with a mixture of meadows and Scots pine forests. Study sites were selected to keep plant communities constant along the gradients (for a detailed description of the gradient see: Moroń et al. 2012). Heavy metal contamination (Zn, Pb, Cd) levels for each site were analyzed (pollen and bee samples) as described by Moroń et al. (2014). For analysing the level in collected samples 5 samples of pollen, 5 male and 5 female individuals were extracted from each trap nest on each site. Due to random events the number of trap nests retrieved from the sites were between 6 and 7, while not all the nests contained developing bees or pollen. Samples were grouped for each site separately for pollen, male and also females. Before analysis, samples were homogenized and dried at 105 °C and analysed for total concentrations of cadmium, lead and zinc with AAnalyst 800 Spectrometer PerkinElmer, Boston, MA, USA). Total fractions of cadmium and lead were analysed using graphite furnace atomic absorption spectrometry (GF-ETAAS), and concentrations of zinc were analysed by flame atomic absorption spectrometry (FAAS). Total metals were extracted with Suprapur HNO3 (Merck, Darmstadt, Germany). Three blank samples were also analysed for background contamination, and analytical precision was assessed with three reference samples with known metal concentrations (lyophilized bovine liver CRM185R, European Commission). Percentage recovery was 80, 86 and 126% for cadmium, lead and zinc, respectively.

**Fig. 1:**
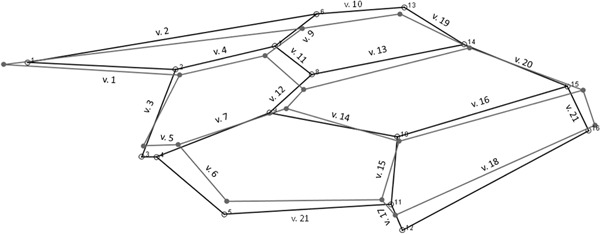
Scheme of 16 (numbers from 1–16) red mason bee wing landmark points and 21 wing veins (v. 1–v. 21) for morphometric measurements and wing venation for males (*black circles*) and females (*open circles*). The wing veins are determined as the distance between the two landmark points measured in a straight line. Differences between the sides were magnified ten times to make them more visible

The heavy metal concentrations in the provisions collected by red mason bees ranged between polluted and unpolluted sites and were found to be positively correlated with concentrations found in top soil (r_s_ = + 0.90, *N* = 5, *p* = 0.083, for further details see Moroń et al. 2012), and also highly correlated with each other on the gradient (for further details see Moroń et al. 2012). However, the levels of cadmium and lead were too low to be detected in bee bodies. Instead, we studied zinc content in the collected provisions correlated with zinc concentration in males and females which were statistically significantly correlated (F(1,53) = 13.21, r^2^ = 0.27, *p* = 0.0006; F(1,53) = 21.30, r^2^ = 0.18, *p* < 0.0001, respectively) for details see Moroń et al. (2014). Therefore to describe the pollution levels on each site concentrations in pollen were used. Concentration of the three metals were highly correlated on the pollution gradients; analysing them separately, when all three were present together on each site was unsubstantiated. We decided to use a single measure of pollution for each site, which describes the site in a more general and overall fashion, rather than analyzing separate models for each metal or choosing one arbitrarily (Moroń et al. 2012). We applied the Princomp procedure implemented in the SAS Institute (2004), and for further analyses we used the first principal component (PC1) score of each trap as a pollution index (Zygmunt et al. 2006; Moroń et al. 2012). A higher PC1 corresponds to higher overall heavy metal contamination of the bees’ provisions (for details see Moroń et al. 2014).

### Trap nests

The bees in our studies originated from the Biodar Bee Breeding Company from Poland. Bees were installed in the field in three successive years (2004–2006) along the heavy metal pollution gradient. For calculation of mean temperatures in the study area, we have used data available from http://www.wunderground.com, using averaged data for the two closest weather stations: EPKT and EPKK in Poland. The stations are located South-West and South-East of the gradient. The year 2005 was found to be the coolest and 2006 the hottest. Mean temperatures in 2004, 2005, 2006 June–August, the period of bee development, were: 17.3 °C, 17.0 °C, 18.0 °C, respectively. At each site, seven trees separated by distances of >200 m were randomly chosen and fitted with one trap nest at a height of ca. 3 m. Each trap consisted of ca. 110, 25 cm long stems of common reed *Phragmites australis* (Cav.) with nodes in the middle. The bundle of stems was covered with a plywood roof and protected from attack by birds with a metal mesh. The mean reed stem diameter was 7.8 ± 1.9 mm (range 6–12 mm). Each year 75 bee cocoons were installed in March/April together with each trap nest. Experimental nests contained the cocoons of *O. bicornis*, whereas control trap nests were empty. On each site four experimental and three control nests were installed. Traps without cocoons were called control and were established to test the assumption of philopatry of red mason bee females (Roulston and Goodell 2011). We found a very low number of emerged individuals per control trap (1.11 ± 1.90; mean ± SD), therefore we recognized the above-mentioned assumptions as justified. Emerging females (due to their fidelity to their natal nest, see also Steffan-Dewenter and Schiele 2005) started their own nests in the artificial trap nest. At the end of the season in October, when all the trap nests contained developed imagos in cocoons ready for overwintering, the nests were taken back to the laboratory and overwintered in a climate chamber at 4 °C. The number of collected trap nests per site per year varied between 6 and 7 because of random events (broken by wind, stolen, etc.). At the end of winter all cocoons were removed from trap nests and transferred to individually marked 1.5-ml plastic tubes. In March/April, when bees would appear naturally, individuals were placed at room temperature, their sex described and body mass weighed after emergence. The same procedure was repeated each season using bees originating from the breeding colony to start the nests at the experimental sites.

### Morphometric measurements

After sexing and measuring body mass, bees were sacrificed by freezing and their wings were collected and scanned for morphometric analysis. Wings were mounted under a Ricoh/Pentax objective with fixed focus (resolution 2800 dpi). Individuals with destroyed or dirty wings were excluded from further analysis. In total 1362 red mason bees (660 females and 702 males) were used. Sixteen landmarks were determined on the forewings and each forewing was automatically measured three times using the DrawWing software (Tofilski 2008). The three measurements are independent of each other and were used to assess measurement error (Palmer 1994; Graham et al. 2010), which was found to be relatively small in all individuals. To assess wing size and shape, first, the configurations of landmarks were aligned using Procrustes superimposition (Dryden and Mardia 1998) in MorphoJ software (Klingenberg 2011). The landmarks were analyzed using methods of geometric morphometrics. These methods allow one to separate size and shape. As a measure of wing size—centroid size (Dryden and Mardia 1998) was used. Shape, on the other hand, was described by Procrustes coordinates, which were scaled to the same size. Wing size asymmetry was measured as the absolute difference between the centroid sizes of the right and the left forewing divided by the mean centroid size and multiplied by 100 (percentage of centroid size difference between left and right wing). A higher value of this measure indicates greater asymmetry between the left and the right wing for an individual. Wing shape asymmetry was measured as the Procrustes distance (measured as Procrustes FA score) between the shapes of the right and the left wing. Centroid sizes, Procrustes coordinates and Procrustes FA scores were calculated in MorphoJ software (Klingenberg 2011). Coordinates of the landmarks were also used to calculate 21 wing vein lengths in MorphoJ. Wing vein asymmetry was calculated by methods of traditional morphometry after extracting the data from MorphoJ. As a measure of wing vein asymmetry, modified index FA2 = |R-L|/((R + L)/2)*100 was used (after Palmer 1994) where R and L are lengths of the right and the left vein, respectively. A higher value of this measure indicates greater asymmetry between the left and the right wing for an individual. To describe the wing asymmetry of red mason bees the following characteristics were analyzed: wing centroid size (hereinafter called “wing size”), percentage of wing centroid size asymmetry (hereinafter called “wing size asymmetry”), Procrustes coordinates (hereinafter called “wing shape”), Procrustes FA scores (hereinafter called “wing shape asymmetry”), wing vein lengths and wing vein length asymmetry.

### Statistical procedures

First, wing size, wing size asymmetry and wing shape asymmetry were compared between males and females using one-way ANOVA, while wing shape was compared using MANOVA. Next, wing size, wing size asymmetry and wing shape asymmetry were compared for each sex separately using ANOVA with site and year as factors. Wing shape was also compared for both sexes separately, using MANOVA with site and year as factors. Wing vein length asymmetries were compared separately for sexes using two-way ANOVA with site and year as factors for each vein. Both ANOVAs and MANOVAs were followed by Spearman rank correlation for pollution level (PC1), when ANOVA or MANOVA indicated significant differences between sites.

Directional asymmetry was tested comparing: (i) wing size of the left and right wings using Student’s t-test for pair wise comparison for each sex, (ii) wing shape compared using MANOVA based on Procrustes coordinates extracted from MorphoJ as a variable and (iii) wing vein lengths compared using one-way ANOVA with sides (left/right) as factor separately for sexes. In all cases when comparing wing vein lengths or their asymmetry measures, a significant *p* value was set at 0.0024 based on Bonferroni’s correction for 21 comparisons.

Wing size was correlated to wing size asymmetry and wing shape using Pearson’s correlation. All statistical comparisons were done using Statistica software v.10 (StatSoft Inc. 2014).

## Results

Females had significantly larger wings than males (Table 1), also their wing shape differed from males (F(28, 2693) = 229.52, *p* < 0.001) (Fig. 1). Asymmetry of wing size and shape in females was on the other hand smaller than in males (Table 1).

**Table 1:** Red mason bee females’ and males’ wing size, wing size asymmetry and wing shape asymmetry compared between sexes using one-way ANOVA

Trait	Mean ± SD		F(1, 1361)	*p*
	Females	Males		
Wing size	1469.3 ± 3.35	1264.0 ± 2.90	2154.8	*p* < 0.0001
Wing size asymmetry	0.205 ± 0.0064	0.229 ± 0.0069	6.36	*p* = 0.0118
Wing shape asymmetry	0.013 ± 0.0001	0.014 ± 0.0002	20.17	*p* < 0.0001

**Fig. 2:**
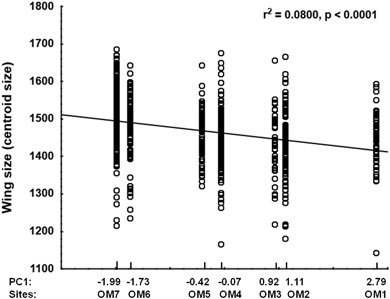
Correlation of forewing size of adult red mason bee females developing on pollen polluted with heavy metals (Zn, Pb, Cd) on 7 sites (OM1–OM7) along a heavy metal pollution gradient. Higher PC1 values (single measure of pollution for each site calculated from the levels of Zn, Pb, and Cd/site) indicate a generally higher pollution level in the pollen provision

Two-way ANOVA of wing size showed, that wing size in females was significantly different between sites, but not between years and also showed an interaction between site and year (Table 2A), while in males only the interaction between year and site was significant. (Table 2A). Sites—based on their PC1 values—were correlated to wing size measures in females using Spearman’s rank correlation and a negative correlation between pollution level and wing size was revealed (r_s_ = −0.0888, *p* < 0.01) (Fig. 2). Two-way ANOVA of wing size asymmetry showed no significant effect or interaction between the test factors, neither in females nor in males. (Table 2B). MANOVA for wing shape showed a significant effect of both pollution and year with year being more significant in both sexes (Table 3). Two-way ANOVA of wing shape asymmetry only showed significant differences between years in both sexes (Table 2C), but no effect of site. Wing venation length asymmetry did not show any correlation to pollution, but some differences were detected between years, namely one vein in females (wing vein 8) and one vein in males (wing vein 10) had different lengths in various years (Table 4).

**Table 2:** Comparison of the difference between red mason bee males and females on a heavy metal pollution gradient in wing size (A), wing size asymmetry (B) and wing shape asymmetry (C) measured in three successive years using two-way ANOVA

Effect	*df*	SS	MS	F	*P*
A Wing size					
Females					
Year	1	7243	7243.01	1.153	NS
Site	4	141,070	35,237.40	5.614	0.0002
Year * site	10	151,590	15,159.04	2.423	0.0081
Males					
Year	1	19,494	19,494.10	3.467	NS
Site	4	28,858	7214.38	1.272	NS
Year * site	10	107,695	10,769.47	1.899	0.0424
B Wing size asymmetry					
Females					
Year	1	0.00604	0.006037	0.220	NS
Site	4	0.22894	0.057235	2.090	NS
Year * site	10	0.30045	0.030045	1.097	NS
Males					
Year	1	0.02464	0.04635	0.752	NS
Site	4	0.08136	0.020340	0.621	NS
Year * site	10	0.42172	0.042172	1.287	NS
C Wing shape asymmetry					
Females					
Year	1	0.000158	0.000170	11.68126	0.0007
Site	4	0.000112	0.000027	1.86904	NS
Year * site	10	0.000191	0.000017	1.15583	NS
Males					
Year	1	0.000069	0.000069	3.871	0.0495
Site	4	0.000030	0.000007	0.414	NS
Year * site	10	0.000071	0.000007	0.396	NS

**Table 3:** Difference between red mason bee males and females on a heavy metal pollution gradient in wing shape measured in three successive years using two-way MANOVA

Effect	*df*	error *df*	F	*P*
Females				
Year	56	2568	5.92	*p* < 0.0001
Site	168	7564	4.47	*p* < 0.0001
Males				
Year	56	2736	4.99	*p* < 0.0001
Site	168	8085	4.76	*p* < 0.0001

**Table 4:** Wing vein length asymmetry in male and female red mason bees developing along a heavy metal pollution gradient and in three successive seasons (2005, 2006, 2007)

Wing vein lenght	Females			Males		
	2005	2006	2007	2005	2006	2007
Vein 1	0.562	0.579	0.585	0.585	0.628	0.580
Vein 2	0.350	0.316	0.322	0.325	0.334	0.357
Vein 3	0.869	0.820	0.692	0.879	0.916	0.833
Vein 4	1.204	1.153	1.172	1.356	1.328	1.321
Vein 5	3.732	3.443	2.959	3.085	3.040	3.431
Vein 6	0.846	0.884	0.766	0.800	1.037	0.907
Vein 7	1.013	0.794	0.657	0.834	0.893	0.807
Vein 8	0.671^a^	0.548^a,b^	0.420^b^	0.509	0.530	0.469
Vein 9	1.810	1.504	1.548	1.937	2.009	2.299
Vein 10	0.886	1.031	0.783	0.992^a,b^	1.183^a^	0.834^b^
Vein 11	1.305	1.432	1.409	1.740	1.553	1.610
Vein 12	2.845	2.302	1.942	3.348	3.312	2.961
Vein 13	0.847	0.821	0.747	0.921	0.783	0.808
Vein 14	0.733	0.685	0.580	0.757	0.774	0.636
Vein 15	1.117	0.917	0.730	0.971	0.950	0.856
Vein 16	1.065	0.548	0.558	0.529	0.471	0.436
Vein 17	4.200	3.226	2.516	3.550	3.706	2.904
Vein 18	0.773	0.556	0.473	0.577	0.596	0.473
Vein 19	1.171	1.531	1.207	1.436	1.439	1.283
Vein 20	1.057	1.169	1.090	1.112	0.925	0.928
Vein 21	4.275	2.673	2.311	3.092	2.807	2.549

Years in each sex were compared using Tukey’s test for uneven sample sizes. Different letters (a, b) indicate significant differences in each sex

In both sexes, left wings (Mean ± SD: females = 1469.6 ± 86.07; males = 1264.4 ± 77.08) were significantly larger than right (Mean ± SD: females = 1468.9 ± 86.41; males = 1263.6 ± 77.00) (females: t(663) = 2.034, *p* = 0.0424; males: t(702) = 3.00, *p* = 0.0028). In both sexes wing shape differed between the left and the right side (females: F(28, 1291) = 2.54, *p* < 0.0001; males F(28, 1375) = 1.81, *p* < 0.006). Analysis of wing venation between sides showed that three veins in females (wing veins 1, 2 and 6) and nine in males (wing veins 1, 2, 6, 8, 12, 14, 15, 17 and 21) differed significantly between the left and the right side and all three in females were the same as in males and also their directionality was the same (wing veins 1 and 2 longer on the right, 6 longer on the left wing) (Table 5).

**Table 5:** Wing vein lengths (mm) of the left and right wings in male and female red mason bees

Wing vein	Females:		*p*	Males:		*p*
	Left wing	Right wing		Left wing	Right wing	
Vein 1	1.245	1.252	<0.001	1.110	1.114	<0.001
Vein 2	2.318	2.324	<0.001	2.005	2.009	<0.001
Vein 3	0.698	0.700	NS	0.583	0.582	NS
Vein 4	0.756	0.754	NS	0.631	0.629	NS
Vein 5	0.178	0.178	NS	0.181	0.180	NS
Vein 6	0.656	0.653	<0.001	0.546	0.543	<0.001
Vein 7	0.928	0.929	NS	0.789	0.789	NS
Vein 8	1.270	1.271	NS	1.077	1.078	0.001
Vein 9	0.394	0.395	NS	0.330	0.331	NS
Vein 10	0.723	0.723	NS	0.637	0.636	NS
Vein 11	0.382	0.382	NS	0.336	0.336	NS
Vein 12	0.351	0.353	NS	0.260	0.262	<0.001
Vein 13	1.268	1.266	NS	1.112	1.110	NS
Vein 14	0.980	0.978	NS	0.826	0.824	<0.001
Vein 15	0.515	0.517	NS	0.434	0.436	<0.001
Vein 16	1.437	1.435	NS	1.254	1.253	NS
Vein 17	0.198	0.197	NS	0.160	0.158	0.002
Vein 18	1.682	1.684	NS	1.458	1.459	NS
Vein 19	0.575	0.573	NS	0.504	0.503	NS
Vein 20	0.907	0.905	NS	0.793	0.791	NS
Vein 21	0.354	0.352	NS	0.288	0.285	<0.001

Sides were compared using Student’s t- test for paired comparison followed by Bonferroni’s correction for 21 comparisons setting significant *p* at 0.0024

In both sexes wing size was positively correlated with body mass (females: r^2^ = 0.7070, *p* < 0.0001; males: r^2^ = 0.5533, *p* < 0.0001). Wing size asymmetry was not correlated to wing size. (females: r^2^ = 0.0021, *p* = NS; males: r^2^ = 0.0010, *p* = NS), while wing shape asymmetry was correlated negatively both in females (r^2^ = 0.0446, *p* < 0.0001) and in males (r^2^ = 0.0173, *p* = 0.0005) to wing size.

## Discussion

Increasing pollution of the environment with cadmium, lead and zinc negatively affected the wing size of red mason bee females, but not of males (Fig. 2). Wing size was also found to be significantly and positively correlated to body size, which is in agreement with the changes in body mass of females reported by Moroń et al. (2014). The same author also found a significant decrease of body mass with increasing levels of pollution in males. The lack of significance for wing size of males in our study—especially considering that a slightly negative trend similar to that in females was present—is most probably caused by the lower sample number used for measurements compared to the study of Moroń et al. (2014).

Exposure to heavy metal pollution did not affect red mason bee wing shape asymmetry, but other environmental factors clearly did. This was visible in the significant differences seen in the red mason bee’s forewing shape asymmetry between years in both sexes. Based on the study of Radmacher and Strohm (2010) showing how temperature might affect the body mass of developing bees, we have calculated the mean temperature in each year for the three most critical months of red mason bee development (June, July and August). At this time most of the bees were already at the prepupa or pupa stage of their development, therefore an effect on wing formation could be expected. Asymmetry measures were the lowest in both sexes when bees were developing in the hottest year (2006) and clearly higher in the two colder years (2004, 2005). We are aware, that such a comparison is not accurate, because only three consecutive years were considered, therefore we do not conclude that such changes in mean temperature between years in our study could on their own significantly affect wing shape asymmetry (for a review of possible effects of temperature on mason bee development see Radmacher and Strohm 2010, 2011; Kierat et al. 2017b). However, it clearly shows how important natural and uncontrolled environmental factors can be during proper wing formation. Similarly, wing vein length asymmetries showed changes between years, but these were not due to pollution. In females, and also in males, one vein length asymmetry showed a significant difference between years (Table 4). The lack of interaction between years and sites confirms that in the case of wing vein length asymmetry as well, an unknown environmental effect simply had a more pronounced effect than pollution itself.

Wing size and shape asymmetries, as well as wing vein length asymmetries, were not affected by pollution, contrary to wing size. These results are in agreement with other studies, showing that both the damselfly *Argia tinctipennis* (Pinto et al. 2012) and the Neotropical orchid bee *Eulaema nigrita* L. (Pinto et al. 2015) caught in degraded or agriculturally intensively managed habitats remained unaffected by environmental stress, although their wing sizes were smaller due to these stressors. In our study, the bees were developing directly under pollution stress, while in Pinto’s study (2015) the test bees were caught in the degraded environment, but there was no information about where they actually developed. Orchid bees can cover large distances and adult individuals present in a certain area can originate and develop in other, distant areas (Pokorny et al. 2015). In our case the origin of bees developing in trap nests on the gradient were undisputable, and heavy metal exposure through provisions consumed was measured (Moroń et al. 2014).

There are a growing number of studies showing that some stressors that are clearly affecting the development of an individual are, however, neutral for wing FA. Some examples are: rearing temperature for honey bees (Jones et al. 2005), malnutrition for honey bees (Szentgyörgyi et al. 2016), climatic and anthropogenic influence on Euglossini bee *Eulaema nigrita* (Silva et al. 2009, but also see *Euglossa pleosticta*—Silva et al. 2009). This negative evidence is in agreement with the suggestion of Beasley et al. (2013), that some of these differences between the various studies may result from the fact that the impact of stress on fluctuating asymmetry seems to be species-, trait- or stressor-specific. Therefore, further studies are needed to unveil the conditions and the traits when FA can be used as a tool for assessing developmental instability.

Wing sizes of red mason bees were found to be different between sexes with females being the larger sex, also having larger wings. Interestingly, directional asymmetry (DA) of wing size was found to be similar in both sexes. DA of size was in favour of the left side in both males and females, contrary to honey bees where right wings are larger (Smith et al. 1997; Schneider et al. 2003; Szentgyörgyi et al. 2016). Difference in shape between the left and the right wing was confirmed by pair-wise comparison of wing venation lengths, describing indirectly also shape. All veins showing directional asymmetry of length in females were also showing DA in males in the same direction. This result indicates similar, but not identical wing venation differences in shape. This is somewhat different and more conservative than in honey bees, where shape and venation differences are more pronounced and less similar between castes (Łopuch and Tofilski 2016). Measuring wing size and shape asymmetry between sexes in both cases, females were found to be more symmetrical than males, suggesting that the sex determination of red mason bees—haplo-diploidity—might affect wing asymmetry levels.

Our results are in agreement with the proposition of Klingenberg et al. (1998) that wing asymmetry is a valuable system to study the evolution of left-right axis establishment in different taxa of flying insects, however, this may not be a good indicator of stress. It was earlier suggested that directional asymmetry is genetically determined and adaptive (Van Valen 1962; Windig and Nylin 1999), therefore, it should not be used as a measure of developmental stability (Palmer and Strobeck 1992). It was even advised that characters that show directional asymmetry should not be used for analysis of fluctuating asymmetry (Palmer and Strobeck 2003). In the present study both size and shape of wing venation showed directional asymmetry. When analyzed individually, some of the wing veins also showed significant directional asymmetry. Therefore, the data presented here about fluctuating asymmetry should be interpreted with care in the light of the directional asymmetry present.

Summarizing, our results showed the lack of a clear impact of heavy metal contamination on FA in the important managed pollinator, the red mason bee, at the same time suggesting the importance of other environmental conditions in the determination of wing morphology. Secondly, our study described and compared, for the first time, the general wing morphology measures of both sexes, showing clear DA of size and shape, which clearly varies from the earlier described DA measures in honey bees.

## References

[CR1] Beasley DAE, Bonisoli-Alquati A, Mousseau TA (2013). The use of fluctuating asymmetry as a measure of environmentally induced developmental instability: a meta-analysis. Ecol Indic.

[CR2] Bellinger DC (2005). Teratogen update: lead and pregnancy. Birth Defects Rest A.

[CR3] Bosch J, Kemp WP (2002). Developing and establishing bee, species as crop pollinators: The example of *Osmia* spp. (Hymenoptera: Megachilidae) and fruit trees. B Entomol Res.

[CR4] Bosch J, Vicens N, Blas M (1993). Análisis de los nidos, de algunos Megachilidae nidificantes en cavidades preestablecidas (Hymenoptera, Apoidea). Orsis.

[CR5] Brückner D (1976). The influence of genetic variability on wing symmetry in honeybees (*Apis mellifera*). Evolution.

[CR6] Calabrese A, Nelson DA (1974). Inhibition of embryonic development of the hard clam, *Mercenaria mercenaria*, by heavy metals. B Environ Contam Tox.

[CR7] Desneux N, Decourtye A, Delpuech JM (2007). The sublethal effects of pesticides on beneficial arthropods. Ann Rev Entomol.

[CR8] Dryden IL, Mardia KV (1998). Statistical shape analysis.

[CR9] EPA (2014) United States Environmental Protecion Agency web page http://www.epa.gov/mercury/effects.htm. Accessed 22 June 2015

[CR10] Francoy TM, de Faria Franco F, Roubik DW (2012). Integrated landmark and outline-based morphometric methods efficiently distinguish species of *Euglossa* (Hymenoptera, Apidae, Euglossini). Apidologie.

[CR11] Francoy TM, Grassi ML, Imperatriz-Fonseca VL, May-Itza WJ, Quezada-Euan JJ (2011). Geometric mophometrics of the wing as a tool for assigning genetic lineages and geographic origin to *Melipona beecheii* (Hymenoptera: Meliponini). Apidologie.

[CR12] Galloway TS, Depledge MH (2001). Immunotoxicity in invertebrates: measurement and ecotoxicological relevance. Ecotoxicology.

[CR13] Gopalakrishnan S, Thilagam H, Raja PV (2008). Comparison of heavy metal toxicity in life stages (spermiotoxicity, egg toxicity, embryotoxicity and larval toxicity) of *Hydroides elegans*. Chemosphere.

[CR14] Graham JH, Raz S, Hel-Or H, Nevo E (2010). Fluctuating asymmetry: methods, theory, and applications. Symmetry.

[CR15] Graham JH, Freeman DC, Emlen JM (1993). Antisymmetry, directional asymmetry, and dynamic morphogenesis. Genetica.

[CR16] Grønkjær P, Sand KM (2003). Fluctuating asymmetry and nutritional condition of Baltic cod (*Gadus morhua*) larvae. Mar Biol.

[CR17] Jones J, Helliwell P, Beekman M, Maleszka R, Oldroyd BP (2005). The effects of rearing temperature on developmental stability and learning and memory in the honey bee. Apis mellifera. J Comp Physiol A.

[CR18] Kierat J, Szentgyörgy H, Woyciechowski M (2017). Orientation inside linear nests by male and female red mason bees (*Osmia bicornis* L., Megachilidae). J Insect Sci.

[CR19] Kierat J, Szentgyörgyi H, Czarnołęski M, Woyciechowski M (2017b) The thermal environment of the nest affects body and cell size in the solitary red mason bee (*Osmia bicornis* L.) J Therm Biol. (in press) doi:10.1016/j.jtherbio.2016.11.008 10.1016/j.jtherbio.2016.11.008 28689719

[CR20] Klingenberg CP (2011). MorphoJ: an integrated software package for geometric morphometrics. Mol Ecol Resour.

[CR21] Klingenberg CP, McIntyre GS, Zaklan SD (1998). Left-right asymmetry of honey bee wings and the evolution of body axes. Proc Roy Soc London B.

[CR22] Krunić MD, Stanisavljević LŽ (2006). Population management in the mason bee species *Osmia cornuta* and *O. rufa* for orchard pollination in Serbia (Hymenoptera: Megachilidae). Entomol Gener.

[CR23] Lima CBS, Nunes LA, Ribeiro MF, de Carvalho CAL (2014). Population structure of *Melipona subnitida* Ducke (Hymenoptera: Apidae: Meliponini) at the southern limit of its distribution based on geometric morphometrics of forewings. Sociobiology.

[CR24] Lyra ML, Hatadani LM, de Azeredo-Espin AML, Klaczko LB (2010). Wing morphometry as a tool for correct identification of primary and secondary new world screwworm fly. B Entomol Res.

[CR25] Łopuch S, Tofilski A (2016). The relationship between asymmetry, size and unusual venation in honey bees (*Apis mellifera*). B Entomol Res.

[CR26] Mather K (1953). Genetical control of stability in development. Heredity.

[CR27] Moroń D, Grześ IM, Skórka P, Szentgyörgyi H, Laskowski R, Potts SG, Woyciecowski M (2014). Survival, reproduction and population growth of the important pollinator bee, *Osmia rufa*, along gradients of heavy metal pollution. Insect Conserv Diver.

[CR28] Moroń D, Grześ IM, Skórka P, Szentgyörgyi H, Laskowski R, Potts SG, Woyciechowski M (2012). Abundance and diversity of wild bees along gradients of heavy metal pollution. J Appl Ecol.

[CR29] Newman MC, Clements WH (2008). Ecotoxicology: A comprehensive treatment.

[CR30] Ondo Zue Abaga N, Alibert P, Dousset S, Savadogo PW, Savadogo M, Sedogo M (2011). Insecticide residues in cotton soils of Burkina Faso and effects of insecticides on fluctuating asymmetry in honey bees (*Apis mellifera* Linnaeus). Chemosphere.

[CR31] Palmer AR, Markow TA (1994). Fluctuating asymmetry analyses: a primer. Developmental instability: its origins and evolutionary implications.

[CR32] Palmer AR, Strobeck C (1992). Fluctuating asymmetry as a measure of developmental stability: implications of non-normal distributions and power of statistical tests. Acta Zool Fenn.

[CR33] Palmer AR, Strobeck C, Polak M (2003). Fluctuating asymmetry analyses revisited. Developmental instability (DI): causes and consequences.

[CR34] Pinto NS, Juen L, Cabette HSR, De Marco P (2012). Fluctuating asymmetry and wing size of *Argia tinctipennis Selys* (Zygoptera: Coenagrionidae) in relation to riparian forest preservation status. Neotrop Entomol.

[CR35] Pinto NS, Silva DP, Rodrigues JG, De Marco P (2015). The size but not the symmetry of the wings of *Eulaema nigrita* Lepeletier (Apidae: Euglossini) is affected by human-disturbed landscapes in the Brazilian Cerrado Savanna. Neotrop Entomol.

[CR36] Pokorny T, Loose D, Dyker G, Quezada-Euán JJG, Eltz T (2015). Dispersal ability of male orchid bees and direct evidence for long-range flights. Apidologie.

[CR37] Radmacher S, Strohm E (2010). Factors affecting offspring body size in the solitary bee *Osmia bicornis* (Hymenoptera, Megachilidae). Apidologie.

[CR38] Radmacher S, Strohm E (2011). Effects of constant and fluctuating temperatures on the development of the solitary bee *Osmia bicornis* (Hymenoptera: Megachilidae). Apidologie.

[CR39] Raw A (1972). The biology of the solitary bee *Osmia rufa* (Megachilidae). T Roy Entomol Soc.

[CR40] Roulston T, Goodell K (2011). The role of resources and risks in regulating wild bee populations. A Rev Entomol.

[CR41] SAS Institute (2004). SAS user’s guide, version 9.1.3.

[CR42] Schneider SS, Leamy J, Lewis A, Degrandi-Hoffman G (2003). The influence of hybridization between African and European honeybees, *Apis mellifera*, on asymmetries in wing size and shape. Evolution.

[CR43] Seidelmann K, Ulbrich K, Mielenz N (2009). Conditional sex allocation in the Red Mason bee, *Osmia rufa*. Behav Ecol Sociobiol.

[CR44] Silva MC, Lomônaco C, Augusto SC, Kerr WE (2009). Climatic and anthropic influence on size and fluctuating asymmetry of Euglossine bees (Hymenoptera, Apidae) in a semideciduous seasonal forest reserve. Genet Mol Res.

[CR45] Smith DR, Crespi BJ, Bookstein FL (1997). Fluctuating asymmetry in the honey bee, *Apis mellifera*: effects of ploidy and hybridization. J Evol Biol.

[CR46] Sorvari J, Rantala LM, Rantala MJ, Hakkarainen H, Eeva T (2007). Heavy metal pollution disturbs immune response in wild ant populations. Environ Pollut.

[CR47] StatSoft, Inc. (2014). Statistica, version 10.

[CR48] Stefanowicz AM, Niklińska M, Laskowski R (2008). Metals affect soil bacterial and fungal functional diversity differently. Environ Toxicol Chem.

[CR49] Steffan-Dewenter I, Schiele S (2005). Nest-site fidelity, body weight and population size of the red mason bee, *Osmia rufa* (Hymenoptera: Megachilidae), evaluated by mark-recapture experiments. Entomol Gener.

[CR50] Stone D, Jepson P, Laskowski R (2002). Trends in detoxification enzymes and heavy metal accumulation in ground beetles (Coleoptera: Carabidae) inhabiting a gradient of pollution. Comp Biochem Physiol C.

[CR51] Szentgyörgyi H, Blinov A, Eremeeva N, Luzyanin L, Grześ IM, Woyciechowski M (2011). Bumblebees (Bombidae) along pollution gradient - Heavy metal accumulation, species diversity, and *Nosema bombi* infection level. Pol J Ecol.

[CR52] Szentgyörgyi H, Czekońska K, Tofilski A (2016). Influence of pollen deprivation on the forewing asymmetry of honeybee workers and drones. Apidologie.

[CR53] Szentgyörgyi H, Woyciechowski M (2013). Cocoon orientation in the nests of red mason bees (*Osmia bicornis*) is affected by cocoon size and available space. Apidologie.

[CR54] Tofilski A (2008). Using geometric morphometrics and standard morphometry to discriminate three honeybee subspecies. Apidologie.

[CR55] Tyler G, Balsberg Påhlsson A-M, Bengtsson G, Bååth E, Tranvik L (1989). Heavy-metal ecology of terrestrial plants, microorganisms and invertebrates. Water Air Soil Pollut.

[CR56] Van Valen L (1962). A study of fluctuating asymmetry. Evolution.

[CR57] Walker CH, Silby RM, Hopkin SP, Peakall DB (2012). Principles of ecotoxicology.

[CR58] Wasielewski O, Giejdasz K, Wojciechowicz T, Skrzypski M (2011). Ovary growth and protein levels in ovary and fat body during adult-wintering period in the red mason bee, *Osmia rufa*. Apidologie.

[CR59] Windig J, Nylin S (1999). Adaptive asymmetry in wing size in the speckled wood butterfly, *Pararge aegeria*?. Proc Roy Soc Lond B..

[CR60] Zygmunt PMS, Maryański M, Laskowski R (2006). Body mass and caloric value of the ground beetle (*Pterostichus oblongopunctatus*) (Coleoptera, Carabidae) along a gradient of heavy metal pollution. Environ Toxicol Chem.

